# Knowledge-based versus deep learning based treatment planning for breast radiotherapy

**DOI:** 10.1016/j.phro.2024.100539

**Published:** 2024-01-20

**Authors:** Daniel Portik, Enrico Clementel, Jérôme Krayenbühl, Nienke Bakx, Nicolaus Andratschke, Coen Hurkmans

**Affiliations:** a European Organisation for Research and Treatment of Cancer (EORTC) Headquarters, Brussels, Belgium; b Department of Radiation Oncology, University Hospital Zürich, University of Zürich, Zürich, Switzerland; c Department of Radiation Oncology, Catharina Hospital Eindhoven, Eindhoven, the Netherlands; d Department of Applied Physics and Department of Electrical Engineering, Technical University Eindhoven, Eindhoven, the Netherlands

## Abstract

**Background and Purpose:**

To improve radiotherapy (RT) planning efficiency and plan quality, knowledge-based planning (KBP) and deep learning (DL) solutions have been developed. We aimed to make a direct comparison of these models for breast cancer planning using the same training, validation, and testing sets.

**Materials and Methods:**

Two KBP models were trained and validated with 90 RT plans for left-sided breast cancer with 15 fractions of 2.6 Gy. The versions either used the full dataset (non-clean model) or a cleaned dataset (clean model), thus eliminating geometric and dosimetric outliers. Results were compared with a DL U-net model (previously trained and validated with the same 90 RT plans) and manually produced RT plans, for the same independent dataset of 15 patients. Clinically relevant dose volume histogram parameters were evaluated according to established consensus criteria.

**Results:**

Both KBP models underestimated the mean heart and lung dose equally 0.4 Gy (0.3–1.1 Gy) and 1.4 Gy (1.1–2.8 Gy) compared to the clinical plans 0.8 Gy (0.5–1.8 Gy) and 1.7 Gy (1.3–3.2 Gy) while in the final calculations the mean lung dose was higher 1.9–2.0 Gy (1.5–3.5 Gy) for both KPB models. The U-Net model resulted in a mean planning target volume dose of 40.7 Gy (40.4–41.3 Gy), slightly higher than the clinical plans 40.5 Gy (40.1–41.0 Gy).

**Conclusions:**

Only small differences were observed between the estimated and final dose calculation and the clinical results for both KPB models and the DL model. With a good set of breast plans, the data cleaning module is not needed and both KPB and DL models lead to clinically acceptable results.

## Highlight

• Both approaches generated comparable deliverable dose results.
• The comparison used the same training dataset for both planning approaches.
• Predicted doses were too optimistic for the knowledge-based planning.

## 1 Introduction

Breast radiotherapy (RT) is a safe and effective element of the oncological treatment of breast cancer [1]. However, some cardiac and lung toxicity is reported which is directly correlated with the dose to the heart and lungs [2], therefore RT plans need to be optimised to spare these organs.

The creation of optimised RT plans is an iterative process involving experts in radiotherapy such as the radiation oncologist, medical physicists and dosimetrists [3]. Automated planning which incorporates dose prediction has been demonstrated as advantageous in terms of time efficiency and plan quality [4], [5], while also has the potential of reducing inter-patient and inter-institution variation [6].

An increasing number of deep learning (DL) models reveal promising results in a variety of clinical scenarios, with a majority using a DL convolutional network architecture, learning from both local and global features to make a voxel-wise prediction of the dose distribution [7], [8], [9], [10]. Dose prediction strategies commonly use a slice-by-slice 2D method, which can lead to errors at the cranio-caudal edges of target volumes. This error motivated the creation of 3D models at the cost of increased computational expense [7], [11], [12].

Knowledge-based planning (KBP) is a type of radiotherapy planning which uses previously acquired knowledge of dose-volume histogram (DVH) parameters in relation to planning target volume and organ at risk locations to create a treatment plan, rather than relying solely on information from the current image used for dose calculation [13]. Several in-house models have been developed for multiple disease sites, with some models also being incorporated within commercial treatment planning software for several clinical scenarios [14], [15], [16], [17]. These models employ geometrical features and the associated dosimetry of plans included in the model library to predict a range of achievable DVH for organs at risk (OAR) of new patients [18]. The prediction range consists of one standard deviation around the mean estimated DVH. Consistency of plans and the range of patient geometries in the model library therefore greatly influence the accuracy of the DVH predictions [18]. The final DVHs from deliverable plans can be compared against the predicted DVHs.

The set of patients in our study, has been previously used to compare two models, a 2D U-net model and a 3D U-Net model [19], showing promising results for automated plan generation. Our comparison enhances the existing literature [20] by adding the predicted doses next to the final calculated ones. To our knowledge, this endeavour represents the first ever comparison between a KBP (geometry-based) solution and a Machine Learning (volumetric) solution for breast cancer radiotherapy encompassing both dose estimations and deliverable dose distributions using the same training set.

## 2 Materials and Methods

### 2.1 Model library

**Fig. 1:**
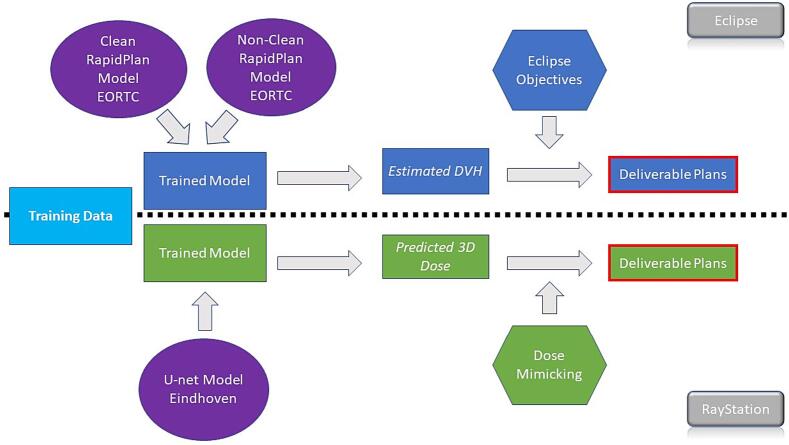
Breakdown of Comparison.

Anonymised data of 105 randomly selected left-sided breast cancer patients, previously used in the work presented by Bakx *et al* [19], were provided by Catharina Hospital Eindhoven, the Netherlands to the European Organisation of Research and Treatment of Cancer (EORTC) headquarters. Patients were initially treated with an Elekta VersaHD (Elekta Ltd, Crawley, UK) machine, the chosen algorithm being Collapsed Cone Convolution. All data were uploaded to Eclipse v15.1 (Varian Medical Systems, Palo Alto, USA) treatment planning software and used to develop a RapidPlan model using RapidPlan v15.6. Similar to the DL model, ninety plans were used to train and validate the models and 15 patients used for testing. (Fig. 1). The models can be shared by submitting a data sharing request to EORTC. Ethical approval was granted by the local ethics committee. This research is conducted on anonymised patient data. All breast treatment plans used a hypofractionated approach, with a prescribed dose of 40 Gy/15fr, scaled so that 95 % of the dose covered 98 % of the planning target volume (PTV). PTV were generated by expanding the clinical target volume by 5 mm followed by a cropping under the skin by 5 mm. The chosen planning technique was tangential IMRT using 6MV or 10MV beams. Dose calculation was performed using Anisotropic Analytical Algorithm version 15.06.

### 2.2 Description of model training

A mix of upper, mean and line objectives were used for the training of the model’s structures of interest: PTV, body, heart and lungs. Line objectives were generated according to estimates, with a line objective being a limit for the dose in a given structure for all volume levels. During the training of the model OAR-preferring line-objectives were selected.

Plans were generated using a Varian TrueBeam machine model, courtesy of University Hospital of Zurich. Due to software constraints, RapidPlan cannot generate estimations for the target volumes, contrary to the RayStation approach. The original paper describing the experience of the DL model comparison used an in-house developed U-net architecture versus a contextual atlas-regression forest model [19].

Of the 105 plans, 72 were used for training, 18 for internal validation and 15 for testing of the KBP model. Although prediction models can be trained with fewer plans, it has been shown that a higher number of plans can result in less dependency on the training set and more accurate predictions [21].

The optimization of the model required the selection and elimination of influential (term used in Varian user manual) data points that reduce the accuracy of the model, with subsequent retraining after each elimination. To identify these points, Cook’s Distance (CD), modified Z-score (mZ), Studentized residual (SR) score and area difference (dA) of estimates [22] metrics were used (Fig. S1).

To avoid overfitting, where the model cannot generalize and fits too closely to the training dataset instead [5], a maximum of 10 % of plans were removed from the original dataset, while also maintaining a large enough training dataset. With elimination of 10 % of plans (7 plans), the coefficient of determination (R^2^) was 0.93 (Table S2).

Two models were created with sequential retraining: a) a Clean Model – with influential data points selection and elimination as described, and b) a Non-Clean Model – using the full original unmodified patient set, as used in the previous DL model (Fig. 1) in the paper published by Bakx *et al* [19].

Within this paper, the terms of *‘calculated’* refer to the actually deliverable plans for both the Clean and Non-Clean models. These are the counterparts of the DL model deliverable plans, called *‘mimicked’* plans in the Bakx paper [19]. *‘Estimated’* values refer to the DVH estimated values for both Clean and Non-Clean models, these being the counterparts of the DL model’s *‘predicted’* doses. The radiotherapy plans used as training data are called ‘*clinical’* plans which were created manually.

### 2.3 Internal validation

For internal validation 18 patients were used, the same patients used in the validation of the DL model. Parameters for comparison were based upon the Dutch national consensus [23] and consistent with those reported by Bakx *et al.* [19].

After several iterations the structures and objectives with priorities that produced the best calculated results were determined using the validation set and remained unchanged at the testing stage (Table S2).

### 2.4 Testing of the model

For the testing of the model 15 patients were used, independent from the validation set. Plan evaluation criteria for the testing phase were as described for internal validation (Table 1). The parameters used in Table S2 in the Supplement were used to generate the DVH estimations and plans for the testing cohort. To assess differences between the Clean and Non-Clean models, averages of mean PTV, PTV D2%, mean heart and mean lung doses were calculated.

**Table 1:** Number of patients used for internal validation (n = 18) and testing (n = 15) achieving a clinical goal. *PTV coverages were still within Dutch national consensus recommendations. RT plans were scaled so that 95 % of the dose covered 98 % of the PTV to ease comparison.

Clinical Goals	Internal Validation		Testing	
Validation Criteria	CleanModel	Non-Clean Model	Clean Model	Non-Clean Model
**PTV: D_mean_ ≥ 40 Gy**	16*	16*	13*	13*
**PTV: D 2 %≤42.8 Gy**	18	18	15	15
**Heart: D_avg_ ≤ 3 Gy**	18	18	15	15
**Lung: D_avg_ ≤ 6 Gy**	18	18	15	15
**Heart: D_avg_ ≤ 2 Gy**	17	17	15	15
**Lung: D_avg_ ≤ 4 Gy**	18	18	15	15
**External - PTV: V107%≤10.0 cm^3^**	17	17	15	15

The DL model has a U-net architecture, and was trained for 800 epochs, with no cross-validation. A sampling scheme (selection of eight slices) was used for each epoch, with each batch containing three patients. Development of the DL model took place with the same dataset of 90 patients for training,18 patients for validation, and 15 patients for testing. For a more detailed overview on the DL training and validation please see Bakx *et al.*[19].

To compare the mean and D2% doses of the different models, Wilcoxon signed-rank (Estimated KBP doses vs. Calculated KBP doses; U-net predicted vs. U-net mimicked doses) and Friedman tests with later pairwise comparisons (Clinical values vs. estimate KBP doses vs. U-net predicted doses; clinical values vs. calculated KBP doses vs. U-net mimicked doses) were used, with a significance level 0.05.

## 3 Results

Regarding the validation process, all clinical goals for PTV D2%, mean heart and mean lung dose were fulfilled. The mean heart dose of < 2 Gy was not achieved in one case; having 2.3 Gy for both KBP models. Although the criteria for mean PTV dose was not reached in two patients (4 plans), the doses achieved were within 0.5 % of the prescribed dose (-0.3 % and −0.2 % for both models). Results of the validation of the Clean and Non-Clean models revealed that both models were unable to meet the goal of Body – PTV V107%≤10.0 cm^3^, for one patient: reaching 15.4 cm^3^ (Clean model) and 15.5 cm^3^ (Non-Clean model) (Table 1).

Results of the model testing phase and comparison with the original clinical plans and U-net predicted and mimicked values are shown in Table 2. All values are reported as median with specified ranges.

**Table 2:** Average and maximum doses in Gy for the clinical, estimated, predicted, calculated, and mimicked plans. All values are reported as median (range). NA - Not Applicable.* a value of 36.8 Gy was erroneously reported in the original paper.

Models		Clinical		Clean Model Estimate		Non-Clean Model Estimate		Clean Model Calculated		Non-Clean Model Calculated		U-net Predicted		U-net Mimicked	
		Mean	D2%	Mean	D2%	Mean	D2%	Mean	D2%	Mean	D2%	Mean	D2%	Mean	D2%
**PTV**	Median Dose (Gy)	40.5	42	NA	NA	NA	NA	40.6	41.6	40.6	41.6	40.4	42.2	40.7	42.3
	Range (Gy)	40.1–41.0	41.6–42.6	NA	NA	NA	NA	39.9–41.6	41.0–42.7	39.9–41.6	41.0–42.7	40.3–40.6	41.9–42.5	40.4–41.3	41.8–43.0
Difference from prescribed dose (%)		1.2	5	NA	NA	NA	NA	1.5	4.2	1.4	4.2	1.1	5.4	1.9	5.9
**Heart**	Median Dose (Gy)	0.8	3.1	0.4	2.4	0.4	2.4	0.8	4	0.8	4	0.9	3.4	0.9	3.2
	Range (Gy)	0.5–1.8	1.5–15.3	0.3–1.1	1.9–15.4	0.3–1.1	1.9–14.8	0.5–1.9	2.2–18.7	0.5–1.9	2.2–18.1	0.7–1.5	2.2–13.2	0.5–1.8	1.6–15.1
**Lung**	Median Dose (Gy)	1.7	24.9	1.4	25.9	1.4	25.9	2	32.1	1.9	31.8	1.8	26.6	1.8	27
	Range (Gy)	1.3–3.2	18.9–36.1	1.1–2.8	18.9–34.2	1.1–2.9	18.9–34.5	1.5–3.5	25.1–35.6	1.5–3.5	25.0–35.6	1.3–2.8	17.3–33.4	1.3–3.2	19–36.5

**Fig. 2:**
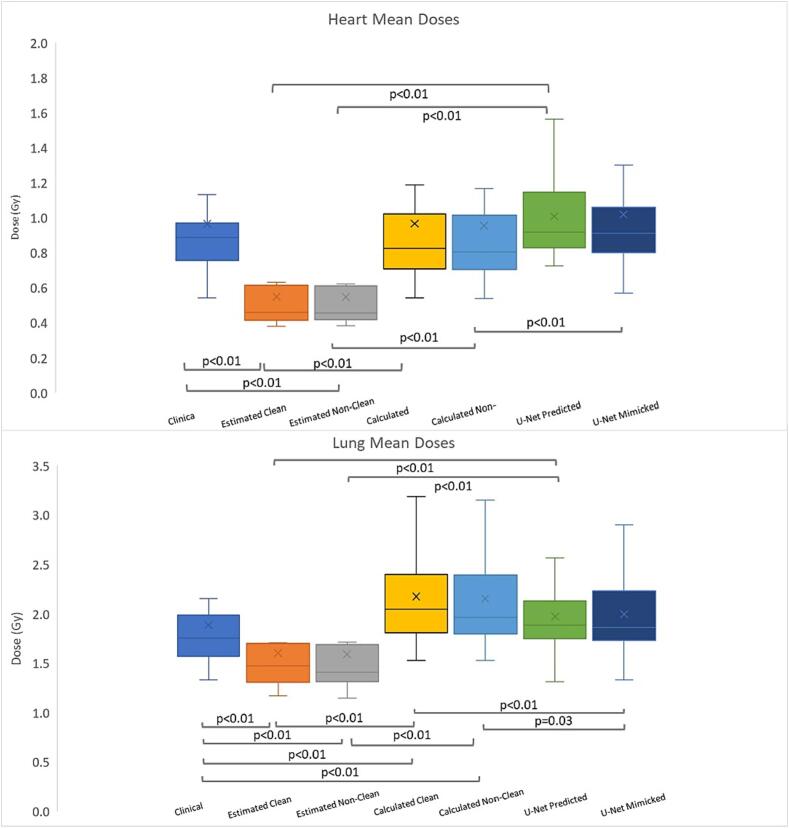
Mean heart and lung doses produced with the different models. Statistically significant pair differences are marked for ease of viewing with respective p values. For full breakdown see Appendix Table A.3a-f.

The estimated mean heart and lung doses for both Clean 0.4 Gy (0.3–1.1 Gy), 1.4 Gy (1.1–2.8 Gy), and Non-Clean model, 0.4 Gy (0.3–1.1 Gy), 1.4 Gy (1.1–2.9 Gy) versions showed statistically significant differences from the clinical, and U-net predicted doses, Fig. 2. Both sets of calculated values for the mean heart doses showed no statistically significant differences compared to the clinical plans, while the Non-Clean calculated values also differed significantly from the mimicked values (Fig. 2). This difference places the Clean model more in alignment with the U-net model for calculated mean heart values. Regarding calculated mean lung doses, 1.9–2.0 Gy (1.5–3.5 Gy), values differed from clinical plans (p < 0.01) and mimicked values 1.8 Gy (1.3–3.2 Gy), p < 0.01 and p = 0.03, for both KBP models (Fig. 2).

The Clean model produced heart D2% estimates, 2.4 Gy (1.9–15.4 Gy), that were statistically significantly different from both clinical, 3.1 Gy (1.5–15.3 Gy), and U-net predicted values, 3.4 Gy (2.2–13.2 Gy), whilst the Non-Clean model showed non-significant difference from the clinical values. The same did not apply to the estimated lung D2% values for both KBP models, these showed statistically non-significant differences from the clinical, 24.9 Gy (18.9–36.1 Gy), and U-net predicted values, 26.6 Gy (17.3–33.4 Gy), Fig. S2 in the Supplement. The calculated heart D2% and lung D2% values of the KPB models were statistically different from the clinical values, while only the Clean KPB models’ values were statistically different from the U-net mimicked values with 3.2 Gy (1.6–15.1 Gy) and 27.0 Gy (19–36.5 Gy). This difference positions the Non-Clean model’s values more in line with the U-net mimicked values, regarding lung D2% values.

The statistically significant differences between estimated values and their calculated counterparts both for the Clean and Non-Clean models were upheld, for both models, in all observed OAR DVH parameters: heart mean, heart D2%, lung mean, and lung D2% values (see Supplement Table S3a–f).

**Fig. 3:**
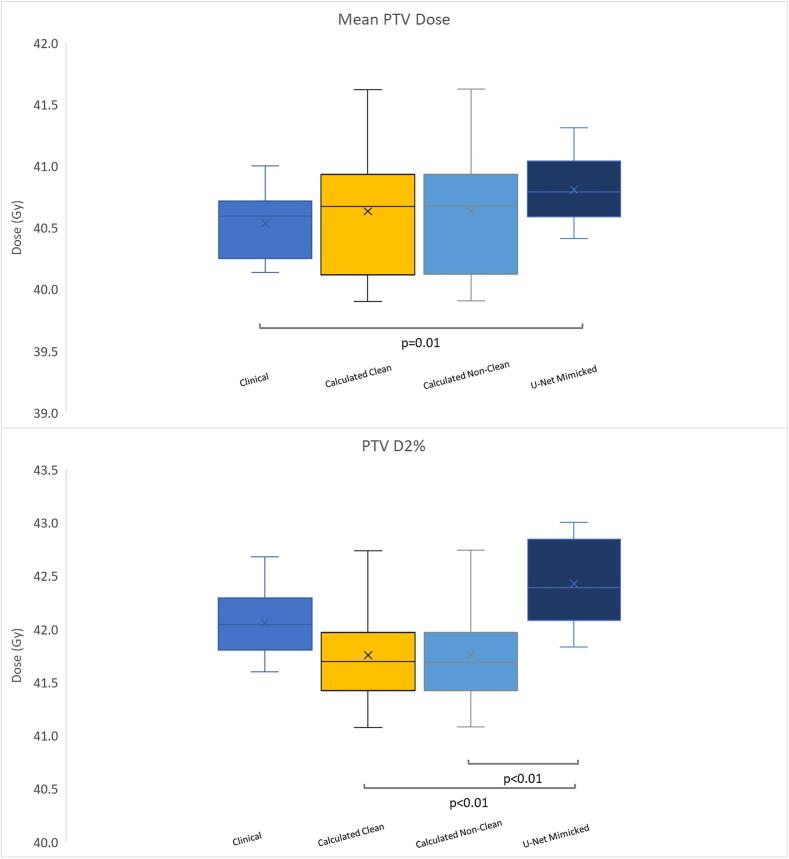
Mean PTV coverages and PTV D2% values produced with the different models. Statistically significant pair differences are marked for ease of viewing with respective p values. For full breakdown see Appendix Table A.3a-f.

The calculated values for both Clean and Non-Clean model versions offered superimposable results for the PTV coverage with a mean of 40.6 Gy (39.9–41.6 Gy), which were statistically non-significant from the U-net mimicked plans and clinical plans, Fig. 3. However, statistically significant differences were present between clinical plans and U-net mimicked values (p = 0.01). Values for the maximum (D2%) PTV values were statistically significantly different for the Clean and Non-Clean models 41.6 Gy (41.0–42.7 Gy) compared to U-net mimicked plans 42.3 Gy (41.8–43 Gy) but not compared to the manual plans. These results point to the conclusion that the KPB models are consistent with the clinical plans, regarding PTV doses.

## 4 Discussion

To our knowledge, this work represents the first comparison of two commercially available solutions for treatment planning automation using both estimated and deliverable doses. Furthermore, this study used two versions of a KBP model to check for the impact of model cleaning procedures. Regarding OARs, we found that the estimated values differed from their calculated counterparts across the board. This overestimation in the KBP model of potential OAR sparing in the estimated doses has been previously reported by Tol *et al* in a study of 20 H&N cancer patients trained using 90 plans. It was noted that the overestimation was present in OARs where the mean doses were > 40 Gy due to the portions of the OAR overlapping with the PTV. This observation is most likely attributable to the horizontal placement of the dose-volume objective at the OAR-PTV overlap [18]. In our testing set of fifteen radiotherapy plans: seven patients presented overlap between Lungs and PTV, while six were borderline and two had no overlap. Furthermore, the choice of the OAR-preferring line objectives was without significant loss of target volume coverage in our KBP models. Similarly, we hypothesize that due to the anatomical particularity of left-sided breast cancer radiotherapy, which often includes close proximity of the heart to the high dose volume, it is difficult for KBP models to accurately make DVH estimations hence the tendency to overestimate heart sparing capabilities. This hypothesis is further supported by the findings of the calculated doses, with the KBP models showing similar mean heart doses compared to the clinical and U-net mimicked doses. This hindrance of models to accurately predict the dose on marginal zones has likewise been described in the literature for DL models [9], [17].

The commercial KBP approach offers an at-hand solution for data cleaning. However, in our study, cleaning the model had a negligible effect, the instances where the Clean and Non-Clean model behaved differently were: mean heart dose calculated Non-Clean vs. U-net mimicked values; heart D2% estimated Clean vs. clinical values; heart D2% calculated Clean vs. U-net mimicked values; lung D2% calculated Clean vs. U-net mimicked values. This difference concerning the D2% values can be explained by the wider range of radiotherapy plans in the Non-Clean model. We acknowledge one possible limitation to our study is the effect of cleaning on the model versions, as we limited our cleaning to approximately 10% of the training set as to not cause overfitting. It's possible that different choices in removing plans could have impacted the outcomes.

The differences noted between the estimated and calculated doses for OARs close to the PTV volume raise the question of whether further investigation is needed. Ideally, the next step would be to compare the different solutions for a higher number of cases with multiple dose level PTV (such as H&N or prostate cancer) and with multiple OAR with both mean and high dose constraints, as several published models have shown robustness over prescriptions with multiple dose levels [9]. The need to increase the number of tested plans is showcased in the heart (mean and D2%), and lung (D2%) values, where we found varying statistically significant differences between the Clean and Non-Clean calculated and mimicked values. Comparing our KBP models’ estimated results with their calculated counterparts, we can state the predicted U-net values are more in line with the U-net mimicked ones, the only statistically significant difference being at the lung D2% value (p = 0.04).

General recommendations for artificial intelligence based applications in RT have been described by Vandewinckele *et al.* [24], wherein they also provide recommendations to improve models for automated treatment planning. Proposed metrics for comparison can include a variety of indices, however the literature is lacking a consensus on how to define the ‘best’ approach. Our approach was based on clinically relevant DVH parameters, relying on the Dutch consensus criteria [23]. We acknowledge that adding a gamma analysis metric [12], calculating isodose volume dice similarity coefficient like used by Ahn *et al.* [20] could be of interest. Castriconi *et al.* [25] showed in a large dataset model that KBP solutions can create similar or even improved values compared to the clinical plans, a finding supported by our results as well, showing DVH values close to their clinical counterparts.

As our results show similar values for both DL and KBP solutions we wish to highlight the recommendation that departments should weigh differences as being clinically relevant or not. Concerning potential limitations, we acknowledge that the use of anisotropic analytical algorithm in our study versus the use of collapsed cone convolution in the works of Kneepkens *et al.* [26] may cause differences in the results. However, our comparison replicates a daily situation with commercially available software in use. It is possible that in the near future, departments will adopt a non-inferiority approach to developing automated planning and using a blinded side-by-side comparison using internal or national guidelines rather than hard cut-off values [27]. Also, the models should be monitored in actual clinical practice as these results can still differ from the test results in a research setting [28].

We believe there are several advantages to our approach. Firstly, the use of the same dataset as for developing a DL model, creating a homogenous radiotherapy plan dataset. Secondly, our models were used for testing both dose prediction and deliverable dose distributions. Thirdly, we could directly compare the Clean and Non-Clean models to the DL model, noting the results of the data cleaning setting our research apart from previous work [20]. Amongst the Clean and Non-Clean models, we were not able to find statistically significant differences for the analysed DVH parameters during the pairwise comparisons.

Estimation of doses is of interest too as a tool for radiotherapy quality assurance, with KBP models having been applied in the H&N cancer setting [18], [29] for this purpose. Accurate estimation of doses via an automatic method would decrease burden on reviewers and enable the addition of quality assurance elements to trials previously overlooked for budgetary or staffing concerns [24]. Although our model versions were capable of providing calculated values in line with clinical criteria, the underestimation noticed in the estimated KBP values draw a cautious lesson for the potential use as semi-automated plan reviews.

In conclusion, our KBP model was able to produce similar deliverable dose results compared to the DL U-net model and the clinical results. Estimated KBP doses were optimistic for mean heart and lung doses. Departments should use the models they have available and prioritize clinical relevance when performing comparisons.

## CRediT authorship contribution statement

**Daniel Portik:** Software, Validation, Methodology, Formal analysis, Investigation, Data curation, Writing – original draft, Writing – review & editing, Visualization, Conceptualization. **Enrico Clementel:** Conceptualization, Methodology, Data curation, Writing – review & editing, Project administration. **Jérôme Krayenbühl:** Validation, Writing – review & editing, Supervision. **Nienke Bakx:** Resources, Data curation, Writing – review & editing, Writing – review & editing, Supervision. **Nicolaus Andratschke:** . **Coen Hurkmans:** Conceptualization, Methodology, Data curation, Writing – review & editing, Supervision, Project administration.

## Declaration of competing interest

The authors declare that they have no known competing financial interests or personal relationships that could have appeared to influence the work reported in this paper.
