# Supplementary material for: Knowledge-based versus deep learning based treatment planning for breast radiotherapy
Source: Phys Imaging Radiat Oncol. 2024 Jan 20;29:100539. doi: 10.1016/j.phro.2024.100539 (PMC10832493; doi:10.1016/j.phro.2024.100539)

Revised supplement with Changes Marked
Click here to view linked References

Supplementary Materials

Table A.1 Model statistics for both Clean and Non-Clean models. R^2 - Coefficient of determination

Model	Model Stage	Number of Patients	Heart R^2	In-Field	Lung R^2
Clean Model	Training	65	0.930	54	0.685
Non-Clean Model	Training	72	0.912	60	0.685

Table A.2 Structures and Objectives with attached Priority used to optimize plans. *Body was used in the optimisation as Body-PTV structure to reduce hotspots outside of PTV

Structure	Objective Type	Volume (%)	Dose	Priority
PTV	Upper	0	101%	200
	Lower	100	99%	200
	Lower	98	95%	0
Body*	Upper	0	102%	350
Heart	Mean	Generated	Generated	0
	Line (preferring OAR)	Generated	Generated	Generated
Lung	Upper	0	5Gy	0
	Mean	Generated	Generated	0
	Line (preferring OAR)	Generated	Generated	Generated

Table A.3a Heatmap of the comparisons for mean heart dose of the different models

**Heart Mean Dose**	Clinical	Estimated Clean	Estimated Non-Clean	Calculated Clean	Calculated Non-Clean	U-net Predicted	U-net Mimicked
Clinical	NA	<0.01	<0.01	1	1	0.81	0.05
Estimated Clean	<0.01	NA	1	<0.01	NA	<0.01	NA
Estimated Non-Clean	<0.01	1	NA	NA	<0.01	<0.01	NA
Calculated Clean	1	<0.01	NA	NA	0.55	NA	0.19
Calculated Non-Clean	1	NA	<0.01	0.55	NA	NA	<0.01
U-net Predicted	0.81	<0.01	<0.01	NA	NA	NA	0.86
U-net Mimicked	0.05	NA	NA	0.19	<0.01	0.86	NA

Table A.3b Heatmap of the comparisons for mean lung dose of the different models

**Lung Mean Dose**	Clinical	Estimated Clean	Estimated Non-Clean	Calculated Clean	Calculated Non-Clean	U-net Predicted	U-net Mimicked
Clinical	NA	<0.01	<0.01	<0.01	<0.01	1	0.62
Estimated Clean	<0.01	NA	1	<0.01	NA	<0.01	NA
Estimated Non-Clean	<0.01	1	NA	NA	<0.01	<0.01	NA
Calculated Clean	<0.01	<0.01	NA	NA	0.26	NA	<0.01
Calculated Non-Clean	<0.01	NA	<0.01	0.26	NA	NA	0.03
U-net Predicted	1	<0.01	<0.01	NA	NA	NA	0.91
U-net Mimicked	0.62	NA	NA	<0.01	<0.01	0.91	NA

Table A.3c Heatmap of the comparisons for heart D2% values of the different models

**Heart D2%**	Clinical	Estimated Clean	Estimated Non-Clean	Calculated Clean	Calculated Non-Clean	U-net Predicted	U-net Mimicked
Clinical	NA	0.03	0.41	<0.01	<0.01	1	0.02
Estimated Clean	0.03	NA	1	<0.01	NA	<0.01	NA
Estimated Non-Clean	0.41	1	NA	NA	<0.01	0.02	NA
Calculated Clean	<0.01	<0.01	NA	NA	0.19	NA	<0.01
Calculated Non-Clean	<0.01	NA	<0.01	0.19	NA	NA	1
U-net Predicted	1	<0.01	0.02	NA	NA	NA	0.49
U-net Mimicked	0.02	NA	NA	<0.01	1	0.49	NA

Table A.3d Heatmap of the comparisons for lung D2% values of the different models

**Lung D2%**	Clinical	Estimated Clean	Estimated Non-Clean	Calculated Clean	Calculated Non-Clean	U-net Predicted	U-net Mimicked
Clinical	NA	0.71	0.71	<0.01	<0.01	1	0.26
Estimated Clean	0.71	NA	1	<0.01	NA	0.16	NA
Estimated Non-Clean	0.71	1	NA	NA	<0.01	0.19	NA
Calculated Clean	<0.01	<0.01	NA	NA	0.13	NA	<0.01
Calculated Non-Clean	<0.01	NA	<0.01	0.13	NA	NA	0.35
U-net Predicted	1	0.16	0.13	NA	NA	NA	0.04
U-net Mimicked	0.26	NA	NA	<0.01	0.35	0.04	NA

Table A.3e Heatmap of the comparisons for mean PTV dose of the different models

**PTV Mean Dose**	Clinical	Calculated Clean	Calculated Non-Clean	U-net Mimicked
Clinical	NA	1	0.19	0.01
Calculated Clean	1	NA	1	0.19
Calculated Non-Clean	0.19	1	NA	1
U-net Mimicked	0.01	0.19	1	NA

Table A.3f Heatmap of the comparisons for PTV D2% values of the different models

**PTV D2%**	Clinical	Calculated Clean	Calculated Non-Clean	U-net Mimicked
Clinical	NA	0.62	1	0.06
Calculated Clean	0.62	NA	1	<0.01
Calculated Non-Clean	1	1	NA	<0.01
U-net Mimicked	0.06	<0.01	<0.01	NA

Figure A.1 –Example of lung geometric outlier – geometric plot (A) and regression plot (B). Selected plan (blue mark) is outside of threshold for overlap volume with the union of targets and also for out-of-field volume.

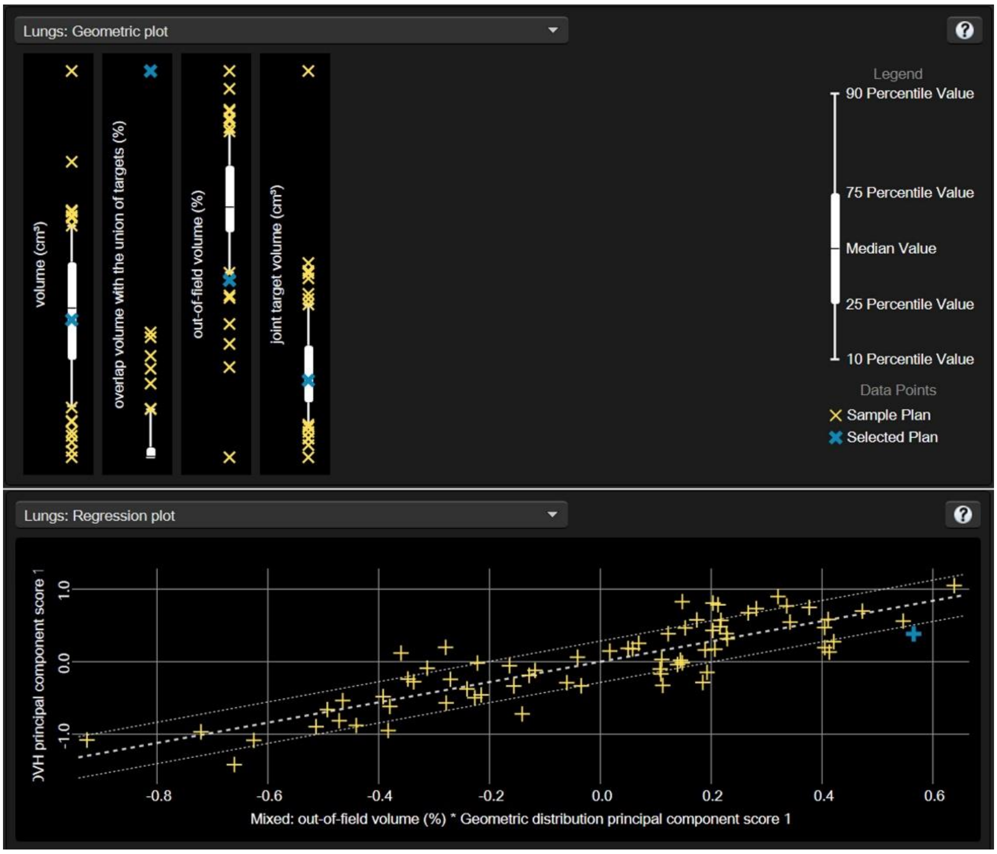

Figure A.2 – Heart & lung D2% values produced with the different models. Statistically significant pair differences are marked for ease of viewing with respective p values. For full breakdown see TableA.3a-f.

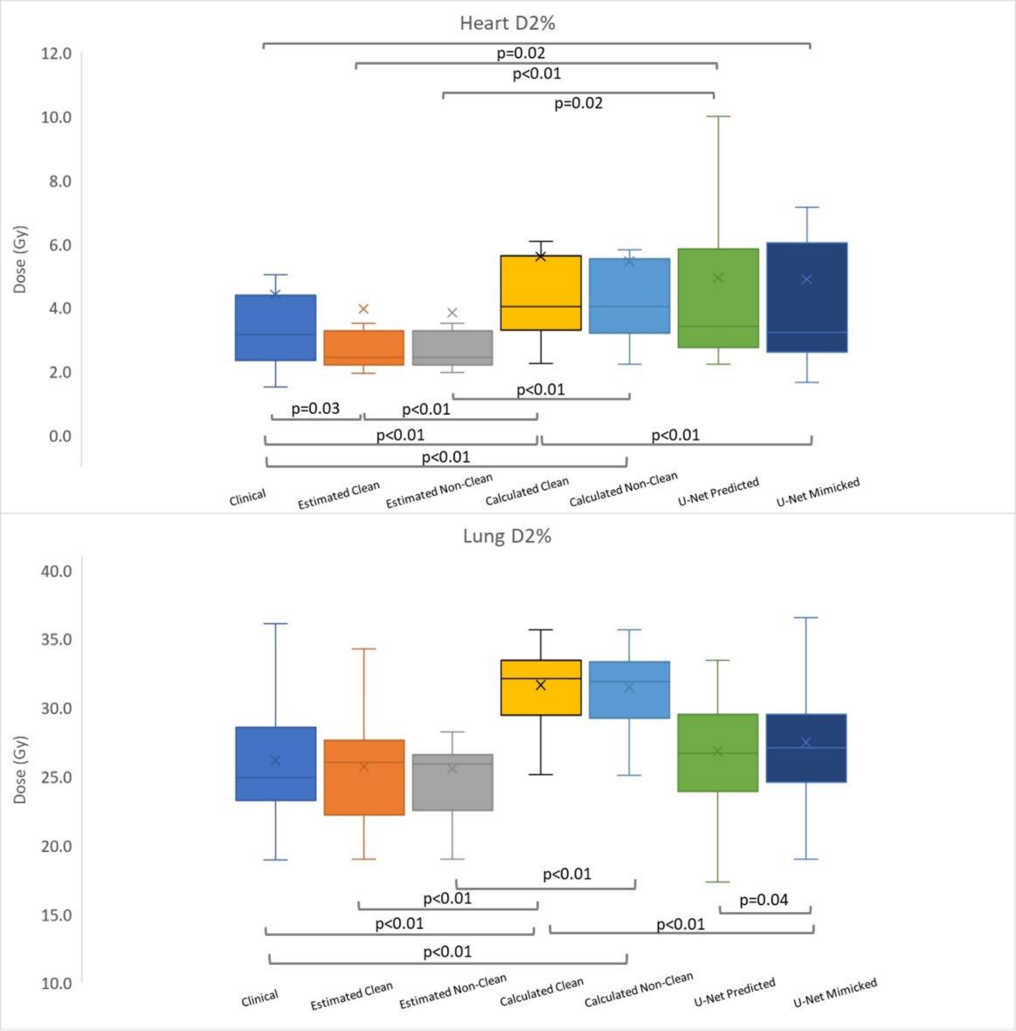

Figure 2

Click here to access/download;Figure;FIG2_COLORED.jpg

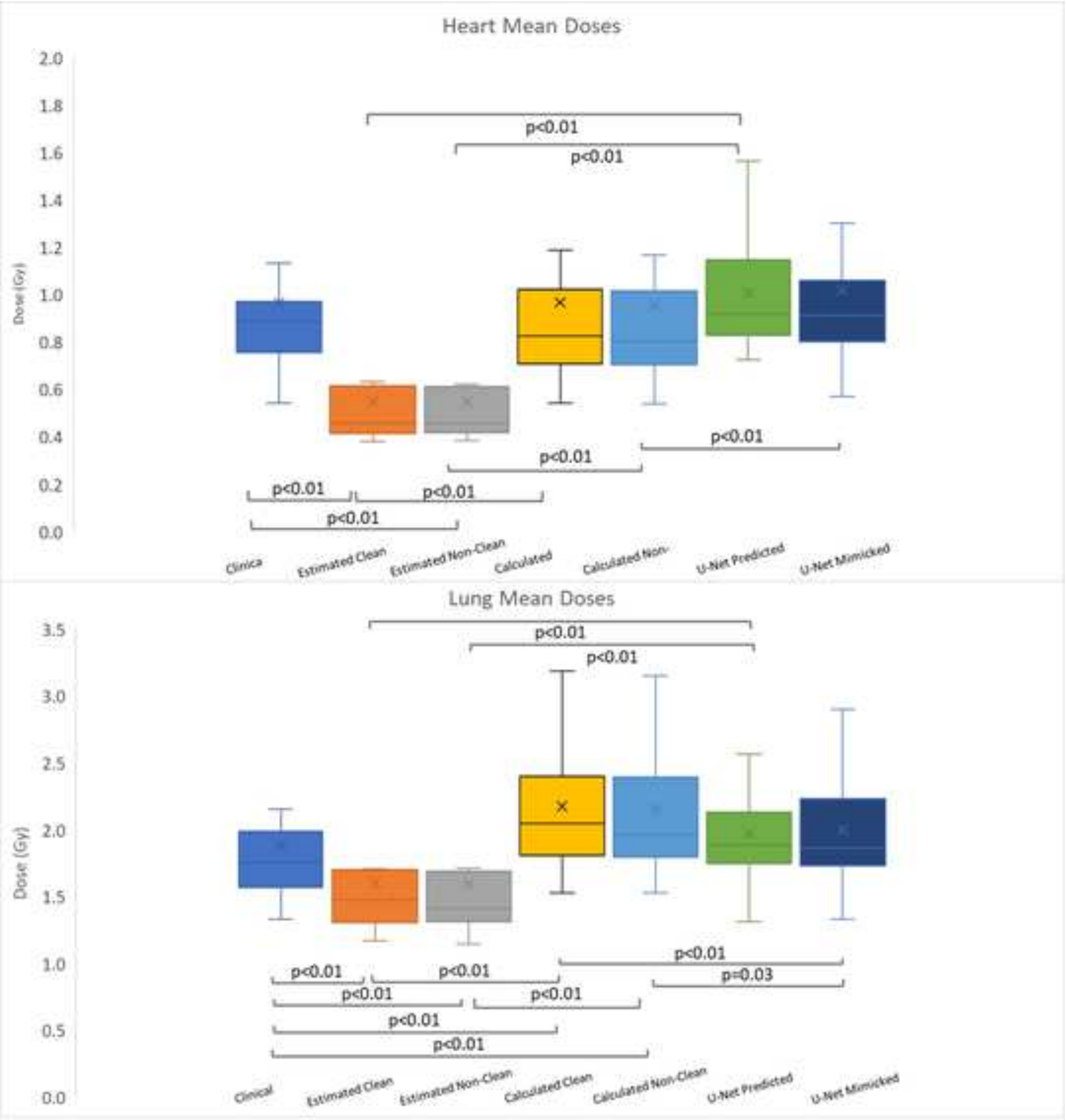

Figure 3 Click here to access/download;Figure;FIG3_COLORED.jpg

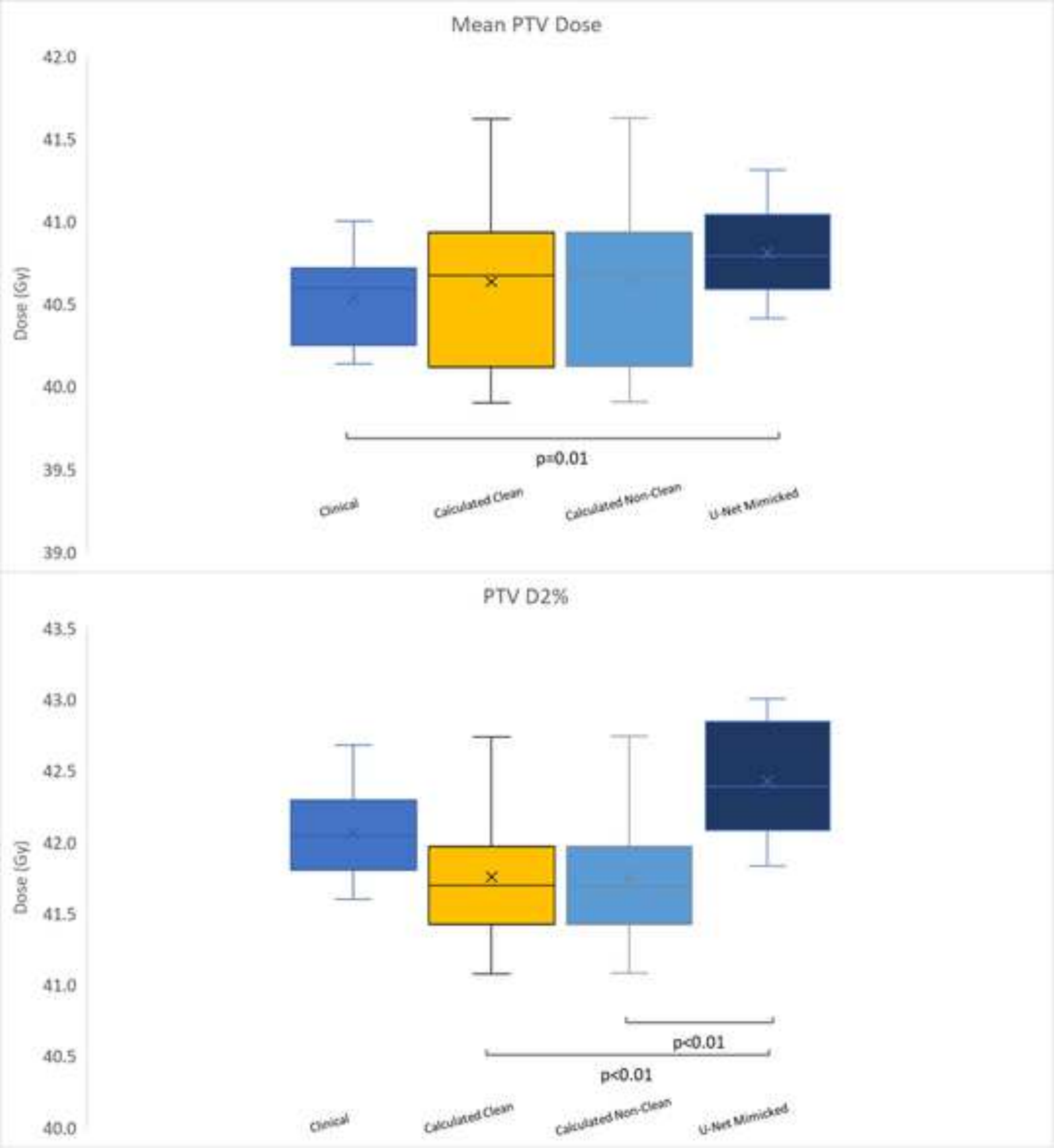

Figure a2

Click here to access/download;Figure;FIGA.2_COLORED.jpg

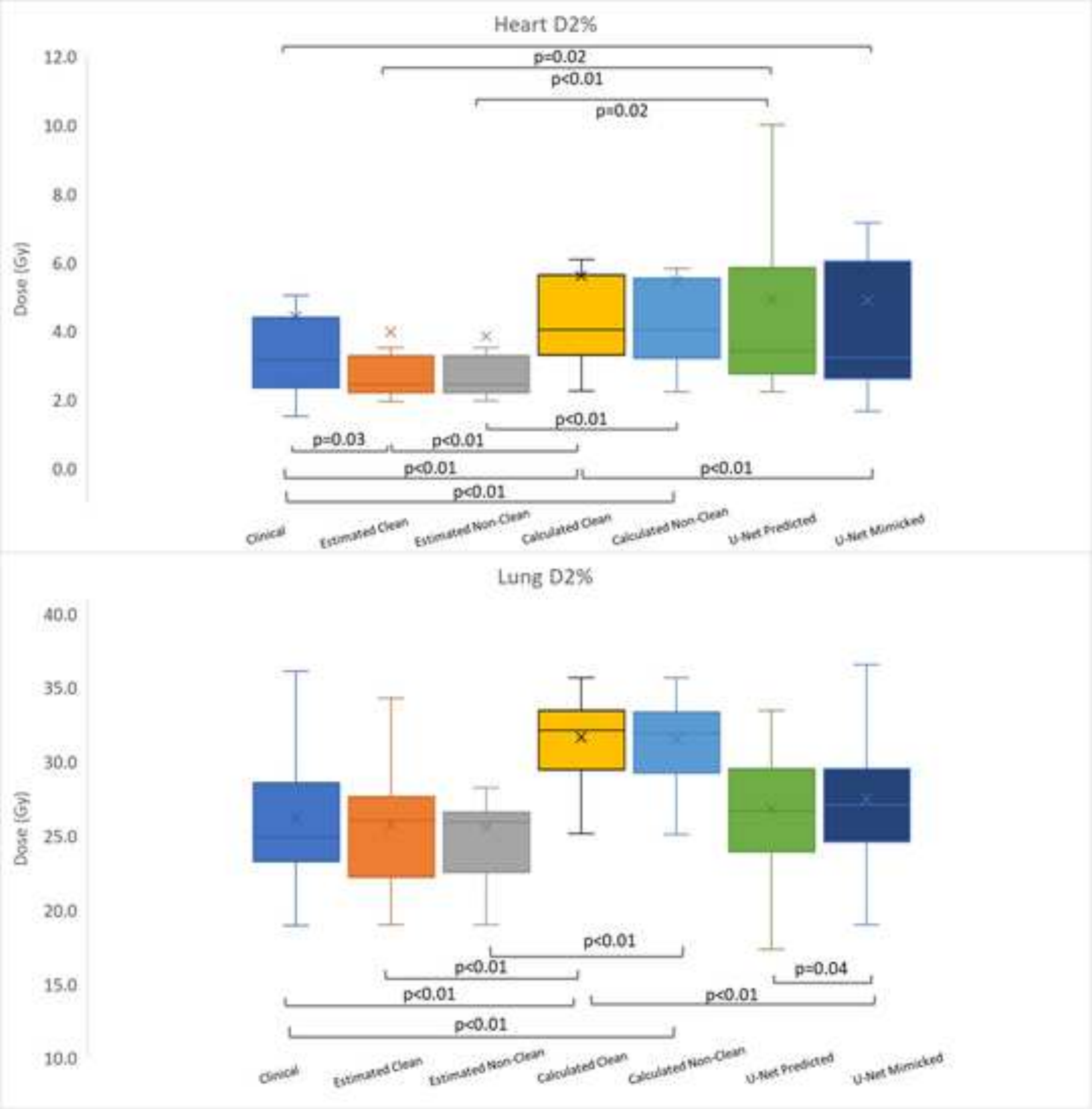

Supplement: Supplementary data 1 [file mmc1.pdf]
